# Early Warning Approach to Identify Positive Cases of SARS-CoV-2 in School Settings in Italy

**DOI:** 10.3390/microorganisms13081775

**Published:** 2025-07-30

**Authors:** Caterina Milli, Cristina Stasi, Francesco Profili, Caterina Silvestri, Martina Pacifici, Michela Baccini, Gian Maria Rossolini, Fabrizia Mealli, Alberto Antonelli, Chiara Chilleri, Fabio Morecchiato, Nicla Giovacchini, Vincenzo Baldo, Maurizio Ruscio, Francesca Malacarne, Francesca Martin, Emanuela Occoni, Rosa Prato, Domenico Martinelli, Leonardo Ascatigno, Francesca Fortunato, Maria Cristina Rota, Fabio Voller

**Affiliations:** 1 Epidemiology Unit, Regional Health Agency of Tuscany, 50141 Florence, Italy; caterina.milli@ars.toscana.it (C.M.); francesco.profili@ars.toscana.it (F.P.); caterina.silvestri@gmail.com (C.S.); martina.pacifici@ars.toscana.it (M.P.); fabio.voller@ars.toscana.it (F.V.); 2 Department of Life Science, Health and Health Professions, Link Campus University, 00165 Roma, Italy; 3 Department of Statistics, Computer Science, Applications (DISIA), University of Florence, 50134 Florence, Italy; michela.baccini@unifi.it; 4 Department of Experimental and Clinical Medicine, University of Florence, 50134 Florence, Italy; gianmaria.rossolini@unifi.it (G.M.R.); alberto.antonelli@unifi.it (A.A.); chiara_chilleri@hotmail.it (C.C.); fmore.po@gmail.com (F.M.); giovacchininicla@gmail.com (N.G.); 5 Microbiology and Virology Unit, Careggi University Hospital, 50134 Florence, Italy; 6 Department of Economics, European University Institute, 50014 Florence, Italy; 7 Department of Cardiac, Thoracic, Vascular Sciences, and Public Health, University of Padua, 35128 Padua, Italy; vincenzo.baldo@unipd.it; 8 Preventive Medicine and Risk Assessment Unit, Azienda Ospedale Università Padova, 35128 Padua, Italy; 9 Division of Laboratory Medicine, University Hospital Giuliano Isontina (ASU GI), 34128 Trieste, Italy; maurizio.ruscio@asugi.sanita.fvg.it (M.R.); francesca.martin@asugi.sanita.fvg.it (F.M.); 10 Public Health Department, University Health Agency Giuliano-Isontina (ASU GI), 34128 Trieste, Italy; francesca.malacarne@asugi.sanita.fvg.it (F.M.); emanuela.occoni@asugi.sanita.fvg.it (E.O.); 11 Department of Medical and Surgical Science, University of Foggia, 71122 Foggia, Italy; rosa.prato@unifg.it (R.P.); domenico.martinelli@unifg.it (D.M.); leonardo.ascatigno@unifg.it (L.A.); 12 Department of Infectious Diseases, Istituto Superiore di Sanità, 00161 Rome, Italy; mariacristina.rota@iss.it

**Keywords:** coronavirus disease, severe acute respiratory syndrome coronavirus 2, screening, salivary test, early-warning method

## Abstract

During the COVID-19 pandemic, some studies suggested that transmission events could originate from schools. This study aimed to evaluate early-warning methods for identifying asymptomatic COVID-19 cases by implementing screening programs in schools. This study was conducted between September 2021 and May 2023, employing a rotation-screening plan for COVID-19 detection on a sample of students aged 14 to 19 years attending secondary schools in the regions of Tuscany, Veneto, Apulia and Friuli-Venezia Giulia. The schools were divided into two groups: experimental and control, with a ratio of 1:2. Two types of molecular salivary tests for SARS-CoV-2 were used to conduct the screening. This study included 16 experimental schools and 32 control schools. Out of 2527 subjects, 11,475 swabs were administrated, with 9177 tests deemed valid for analysis (a 20% loss of tests). Among these, 89 subjects (3.5%) tested positive. In control schools, 1895 subjects (6.5%) tested positive for SARS-CoV-2. This study recorded peaks in infections during the winter and autumn months, consistent with patterns observed in the general population. Beginning in September 2022, a shift occurred, with 2.6% of positive cases reported in the case schools compared to 0.3% in the control schools. Initially, most cases of COVID-19 were detected in the control schools; however, as the pandemic emergency phase concluded, cases were primarily identified through active screening in experimental schools. Although student participation in the active screening campaign was low during the project’s extension phase, this approach was efficacious in the early identification of positive cases.

## 1. Introduction

On 11 March 2020, the World Health Organization (WHO) [1] declared the epidemic of the new coronavirus disease (COVID-19) a pandemic, which had spread globally by June 2020, with more than 7 million confirmed cases and over 400,000 deaths [2]. Similarly, in 2020, the SARS-CoV-2 outbreak had a severe impact on the Italian population.

Briefly, to understand the Italian context of the pandemic, some important dates should be mentioned. On January 31, the Minister of Health in Italy signed an ordinance that closed flights to and from China [3]. However, the virus was already circulating silently within the country, and the first cases were reported at the end of January 2020 [4]. Subsequently, the number of infections rapidly increased. On 5 March 2020, schools and universities were closed (Decree from the President of the Council of Ministers of 4 March 2020) [5]. Italy entered a nationwide lockdown on 9 March 2020 (Decree of the President of the Council of Ministers of 9 March 2020) [6]. On 20 March 2020, when the country had already entered a nationwide lockdown, the Istituto Superiore di Sanità (ISS) and the Istituto Nazionale di Statistica (ISTAT) reported the peak number of COVID-19 cases since the beginning of the pandemic [7]. Only in June 2020 (Decree of the President of the Council of Ministers of 11 June 2020) was movement between regions permitted [8]. In November 2020, the new wave of COVID-19 infections began, leading to restrictions, differentiated based on the risk found in the different Italian regions [9]. On 27 December 2020, the vaccination campaign against COVID-19 officially began across Europe [10]. On 28 June 2021 [11] in the “white zone”, the obligation to wear a mask outdoors lapsed, and the parameters for risk assessment in Italian regions changed while the green pass was developed [12]. On 11 January 2021 (Decree of the President of the Council of Ministers of 5 January 2021), in-person school resumed in high schools [13].

The rapid increase in the number of COVID-19 cases, hospitalizations and deaths during the first months of the pandemic led Italy to experience an extraordinary strain on the national health system (SSN), causing an unprecedented crisis due to the increasing pressure on hospitals and medical staff [14].

One of the first actions implemented by the Italian government to reduce the transmission of SARS-CoV-2 was to arrange the closure of schools, extremely favorable places for the virus to spread. For this reason, in March 2020, the Italian government implemented drastic measures attempting to contain the spread of the virus, with a particular focus on living environments that could most favor the circulation of COVID-19. The decision was made in March 2020, leading to a rapid transition from face-to-face learning to distance education (DAD) and new challenges for teachers and students to adapt to online learning [15].

Further studies [16] reported that less than 5% of COVID-19 cases recorded in EU countries involved individuals under the age of 18 years. Other studies have suggested that SARS-CoV-2 transmission among children within school settings has not been reported to pose a high risk for the adult population [17]. These results could be attributed to a tendency to detect only symptomatic cases, underestimating the number of asymptomatic cases. However, Meuris et al. [18], in a study evaluating the role of children in pandemic transmission, suggested that most transmission events originated within schools. Although many studies have investigated the transmission of SARS-CoV-2 in school settings [19,20,21,22], the data on the effectiveness of early screening strategies for the asymptomatic secondary school population in Italy remain limited [22,23,24,25]. Specifically, between September 2020 and March 2021, Bert et al. [22] conducted a study among 13,283 pediatric patients who underwent a swab in four different hospital settings (school hot spot, emergency department, day hospital setting and hospital wards). The study described a SARS-CoV-2 testing model based on the spontaneous presentation of pediatric patients without a medical prescription and investigated its appropriateness. They found a high incidence of pediatric patients testing positive for SARS-CoV-2 through hot spot detection at the school compared with other settings. Another Italian study [23], conducted in the period September to 15 December 2021, aimed to assess the spread of SARS-CoV-2 within schools in Modena province and the influence of vaccination coverage in these settings. After the identification of an index case, 594 classes were followed by the Public Health Department for a total of 13,934 subjects (1400 teachers and 12,534 students) tested for SARS-CoV-2. In the study population that underwent the swab, 1373 (9.9%) were identified as confirmed cases (10.6% of teachers and 9.8% of students). Among the 594 index cases, 101 (17.0%) were teachers and 493 (83.0%) were students; among the 779 secondary cases, 47 (6.0%) were teachers and 732 (94.0%) students, respectively. When the authors considered the population aged ≥12 years for whom vaccination was regularly offered, 68.5% of the population was vaccinated. Only 11.3% of confirmed cases and 7.8% of secondary cases were vaccinated.

Farina et al. [24] describe the first study conducted in Italy in children of the Piedmont Region (Northern Italy) from January to March 2021. Overall, 69% of the schools and 19.5% of the students in the target population had participated in the screening program, identifying 114 positive cases of SARS-CoV-2. Of these, 69 positive students had been identified before the start of distance learning. Bonaccorsi et al. [25] tested a total of 18,414 students and school personnel at primary and middle schools in Florence, showing that the spread of SARS-CoV-2 in the school setting was low (0.08%) in Florence during the period from 16 November 2020 to 12 February 2021.

Therefore, the objective of this study was to evaluate the effectiveness of innovative early-warning approaches for identifying asymptomatic SARS-CoV-2 cases through the implementation of screening procedures within the school population. Specifically, this study evaluates as follows: (1) the adherence to screening; (2) the incidence of SARS-CoV-2 cases in experimental schools (assessing the early-warning approaches) compared to control schools; (3) a description of the temporal profile of the epidemic spread in the experimental schools compared to that observed in the control schools.

## 2. Methods

### 2.1. Study Design

This study, conducted between September 2021 and May 2023, involved the implementation of a screening-rotation plan on a sample of the student population aged 14–19 years, attending secondary schools in the regions of Tuscany, Veneto, Apulia and Friuli-Venezia Giulia.

In both the experimental and control schools included in this study, the age distribution of students followed the typical pattern observed in Italian secondary schools. Most students in the first year of secondary school are typically aged between 14 and 15 years old. In the second year, they are usually between 15 and 16 years old. In the third year, students range from 16 to 17 years old. By the fourth year, students are typically between 17 and 18 years old, and in the fifth year, they are usually 18 or 19 years old. A small proportion of students in each grade were outside the standard age range, mainly due to grade repetition or non-linear educational trajectories. This distribution was consistent across both groups of schools, ensuring comparable age profiles in the education settings under investigation.

This study was based on a screening plan with repeated in-class tests (at regular intervals every 15 days) on all participating students, considering the classes as the detection unit of interest for possible outbreaks within the school.

This project was divided into three phases, corresponding to the pilot phase (Phase 1), the experimental (Phase 2) and the extension of the experimental phase (Phase 2.0).

The pilot phase (Phase 0) was conducted from May 2021 until the end of the school year (June 2021) and preliminarily validated the proposed procedures.

Phase 2 started on 18 October 2021 and ended on 15 March 2022.

Taking into account the field feasibility of implementing a rotational screening plan, demonstrated through Phase 1 of the project, Phase 2 focused on evaluating the effectiveness of a rotational screening procedure within the class, testing students every two weeks.

Phase 2.0 was an extension of the experimental one (Phase 2), involving the testing of participating students every 15 days. It began on 15 October 2022 and ended on 31 March 2023.

### 2.2. Procedures in the Experimental Schools

Starting from the idea that the class represents an aggregate of subjects who share the same space for long periods during the day, it is the unit of interest for detecting possible outbreaks of infection within the school. The project considered screening plans based on repeated in-class assessments, conducted regularly on a rotating basis every 15 days for students in experimental schools. In each region, at least two schools were selected for screening activities, while four schools were designated as control schools (1:2 ratio). The project also included a referent within each experimental school, as well as the training of students who had given informed consent for the self-administration of molecular salivary tests.

The screening population was identified within the enrolled schools through informed consent signed by the students or parents/guardians in the case of minors. In control schools, the entire student population was considered during the study period.

Thus, overall, this study was conducted on approximately 3000 students in a ratio of 1:2 for the experimental and control groups respectively (approximately 1000 for active screening and 2000 for control students).

The screening plan included repeated in-class tests for COVID-19, conducted in rotation every 15 days on all participating subjects. Different types of molecular salivary tests for SARS-CoV-2 infection detection were used to conduct the screening.

Screening adherence, COVID-19 case incidence, and the temporal profile of the epidemic’s spread were assessed and compared between the experimental and control schools.

### 2.3. Procedures by Region

Based on the territories involved by the participating regions, the reference laboratories for the analysis activity were selected. Specifically:

Friuli-Venezia Giulia (FVG) involved the Complex Structure of Microbiology and Virology of Cattinara Hospital (Trieste, Italy);

Apulia involved the Operating Unit of Microbiology and Virology, University Hospital Trust Policlinico di Foggia;

Tuscany involved the Department of Experimental and Clinical Medicine of the University of Florence and specifically the Laboratory of Microbiology and Virology of the University Hospital of Careggi (Florence, Italy); Veneto involved the Laboratory of the Department of Medicine of the University of Padua (DIMED).

The regions of Tuscany used SARS-CoV-2 Panther (Hologic, Manchester, UK) or Allplex SARS-CoV-2 (Seegene Inc., Seoul, Republic of Korea). The Aptima^®^ SARS-CoV-2 Assay is a nucleic acid amplification in vitro diagnostic test for the qualitative detection of RNA (the limit of detection was determined to be 0.01 TCID_50_/mL in the test sample, Hologic documentation); the Allplex™ SARS-CoV-2 Assay is a multiplex real-time PCR assay to detect 4 target genes of SARS-CoV-2 (Limit of detection: 50 copies per reaction, Seegene documentation); Friuli-Venzia Giulia used Lollisponge (Copan, Brescia, Italy) molecular salivary tests was tested for SARS-CoV-2 detection with the PCR method [26]. Veneto used Salivette molecular salivary tests (SARSTEDT AG & Co., Nümbrecht, Germany) using the molecular biological PCR method. Puglia used RT-PCR on the Seegene machine (Seegene Inc., Seoul, Republic of Korea) and antigen test on Fujirebio equipment (Fujirebio Holdings Inc., Tokyo, Japan).

Following the indications provided by the European Centre for Disease Prevention and Control (ECDC) [27], as well as the new provisions transmitted by the Ministry of Health and the Istituto Superiore di Sanità (ISS) [28], the operational phase of the protocol (school year 2021/2022) focused on the use of self-administered molecular salivary tests. This approach aimed to reduce the invasiveness of the nasopharyngeal test, encouraging student compliance at a time when the regulations imposed by the “green pass” required people in contact with those positive to be tested for SARS-CoV-2.

To reduce errors in the self-administration procedure, each region organised training days for each school, using specifically dedicated operators who illustrated and supported the entire screening process, in some cases also using video material.

For the Tuscany region, the percentages of people aged 14–19 years who had taken one dose of vaccine in the previous six months at the end of 2021, 2022 and in April 2023 were reported. These data are not available for the other regions involved in this study. We have cited the vaccination coverage data published by the Ministry of Health as of 23 September 2023 [29].

### 2.4. Data Management

To assess the effectiveness of the school-based rotating screening plan, experimental schools were compared to the control schools. To this end, the control schools used an ad hoc platform built for this study, in which the contact persons reported positive cases notified to the school during the study period by families of students. The cases were registered by entering anonymous records and showing only the date of notification of the positive test, the class and the year of birth of the positive students. On the 30th of each month, the selected laboratories sent the Regional Health Agency of Tuscany individual data relating to the tests (positive/negative results and viral load) carried out as part of the project. The Regional Health Agency of Tuscany extracted the compiled database of participating schools (both experimental and control) every month. The Regional Health Agency of Tuscany also extracted and analyzed the data.

### 2.5. Statistical Analysis

The analysis was conducted by employing both frequency (expressed as a percentage) and absolute number (N) as descriptive statistical tools, in order to illustrate and compare the distribution patterns of the variables under consideration, along with the relevant sociodemographic characteristics, across the different phases of the COVID-19 pandemic.

To test the hypothesis that the two trends were similar, a Poisson regression model was applied, with the number of positive subjects as the outcome and the number of valid swabs (for experimental schools) or total enrolled students (for control schools) as the denominator. The model included the type of school (control or experimental, categorical variable), time (month and year, continuous variable), and the interaction between these two covariates. The evaluation involves the interaction of these parameters using a model where a result of 1 indicates the trends are similar, and a result different from 1 indicates they are not. An adjusted model, considering region and school grade (from 1st to 5th grade), was also applied alongside the basic model. 

## 3. Results

### 3.1. Study Population

This study, conducted over a period spanning from September 2021 to May 2023, focused on the implementation of an active screening program for COVID-19 among students aged 14–19 years. The target population consisted of adolescents attending secondary schools located in four Italian regions: Tuscany, Veneto, Apulia and Friuli-Venezia Giulia. These regions were selected to ensure geographic diversity and represent different epidemiological and organizational contexts within the country.

A total of 48 secondary schools took part in this study. Of these, 16 schools were actively involved in the COVID-19 screening intervention, while the remaining 32 served as control schools. The control schools did not implement the screening procedures but were monitored for comparison purposes. This design allowed for an evaluation of the screening program’s effectiveness in an educational setting. The full list and characteristics of participating schools are presented in Table 1.

This study involved approximately 3000 students, who were assigned to either the experimental or control group following a 1:2 ratio. Specifically, around 1000 students were included in the active screening group, where regular testing procedures were implemented, while approximately 2000 students were part of the control group.

### 3.2. Adherence to Screening and Incidence of SARS-CoV-2 Cases

Out of the total student population attending the selected schools for the screening program (N = 17,580), 2527 students formally agreed to take part in this study. This corresponds to a participation rate of 14.4%, reflecting a moderate level of engagement with the initiative within the target schools.

Participation levels, however, varied significantly over time. During the first school year of implementation (2021–2022), a total of 2233 students adhered to the screening protocol. This number declined sharply in the second year of this study (2022–2023), with only 294 students continuing to participate in the program. Table 2 and Table 3 provide a detailed breakdown of student participation by school year and region.

These figures highlight the challenges of sustaining long-term engagement in public health interventions within school settings, especially in the absence of mandatory measures or perceived immediate risk.

**Figure 1:**
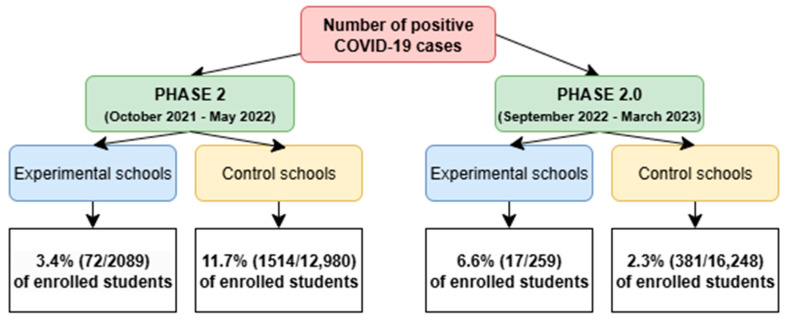
Flowchart of SARS-CoV-2 screening in experimental and control schools. Legend: Number and frequencies of positive cases of COVID-19 in experimental schools and control schools during Phase 2 (October 2021–May 2022) and Phase 2.0 (September 2022–March 2023).

During the entire study period, the results show that among the participant students, 2348 students recorded at least 1 valid swab for analysis. In addition, 11,475 swabs for SARS-CoV-2 detection were taken from the sample. Among them, only 9177 swabs were valid for analysis due to insufficient sample volume, showing a sample loss of 20% (Figure 1).

The gender distribution of the swabs taken shows a higher presence of females in the sample (females = 6201; males = 5274) but without any noticeable gender differences. Overall, 89 swabs tested positive during the entire study period, 3.5% of the screening sample. On the contrary, the positives detected within the schools undergoing testing outside the screening trial represented 10% of the total number of students enrolled (N = 1763).

### 3.3. Temporal Profile of the Epidemic Spread Within the Schools

**Figure 2:**
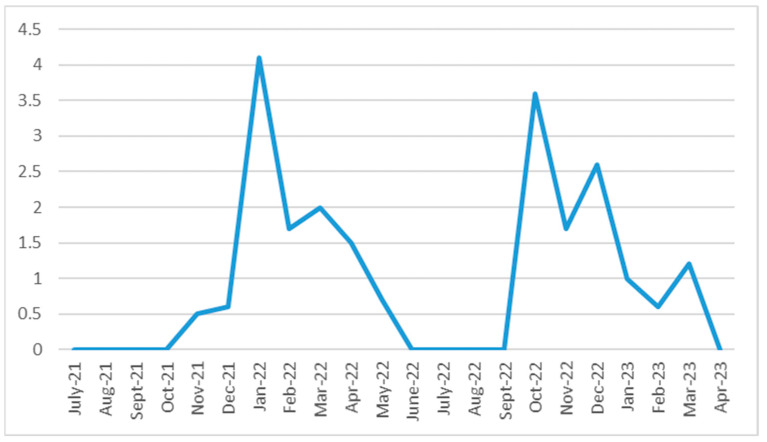
SARS-CoV-2 cases identified from saliva screening in experimental schools in the study period, September 2021–May 2023. Legend: Number of SARS-CoV-2 cases detected through saliva-based molecular screening conducted in participating experimental schools during the study period (September 2021–May 2023), stratified by time interval (monthly). Each dot represents the number of reported positive cases within the corresponding time frame, calculated in relation to the total number of students enrolled in the control schools. The enrolled population included students aged 14 to 19 years.

The study period from September 2021 to May 2023 highlighted two peaks of positive infections identified through screening (Figure 2).

The figure shows a progressive decrease in screening in experimental schools in COVID-19 cases over time, like the general population.

**Figure 3:**
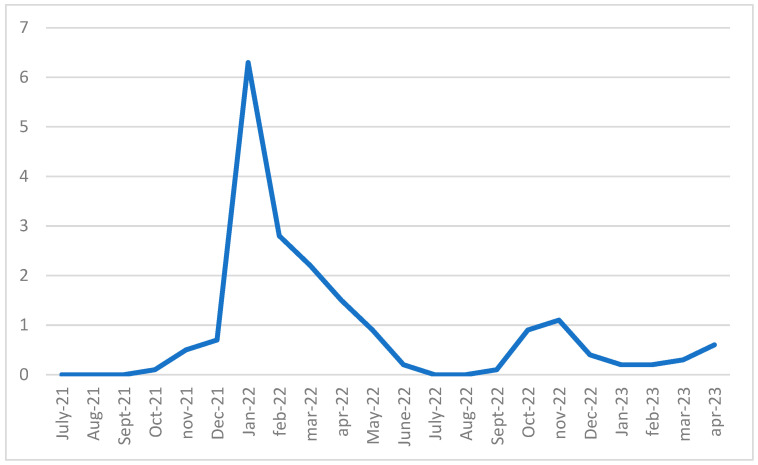
Notified cases of SARS-CoV-2 in control schools in the study period September 2021-May 2023. Legend: Number of SARS-CoV-2 cases officially notified in control schools during the study period (September 2021–May 2023). Data are stratified into monthly intervals. Each dot represents the number of reported positive cases within the corresponding timeframe, connected to illustrate the temporal trend. The enrolled population included students aged 14 to 19 years.

During the same observation period, data on SARS-CoV-2 positive cases were prospectively collected from the control schools enrolled in this study. In total, 29,228 students participated in the surveillance, among whom 6.5% (N = 1895) were confirmed positive for SARS-CoV-2. The highest incidence of infections was observed between January 2022 and March 2022, corresponding to the peak period in the Italian general population (Figure 3).

The figure shows a progressive decrease in screening in control schools in COVID-19 cases over time, like the general population. In control schools, the curve remains constant at very low values.

In January 2022, in schools undergoing screening, the risk of testing positive increased, showing 4% of positive students out of the total number of adherents. Similarly, in the same month, 5.1% of students enrolled in the control schools tested positive for COVID-19.

As this study continues, reports of positivity in control schools tend to decrease and remain stable at very low values. In contrast, in the screened schools, new SARS-CoV-2 positivity peaks were registered in the screened schools during October and December 2022, with a rate of 3.6% and 2.6% of the enrolled sample, respectively.

In addition, the two trends for experimental and control schools were different, and the risk of a positive swab was higher in the experimental schools compared to control schools. In fact, the interaction parameter (school type × time) was equal to 1.26 (1.21–1.32, *p*-value < 0.001) in the crude model and 1.34 (1.29–1.40, *p*-value < 0.001) in the adjusted model, respectively.

Regarding anti-SARS-CoV-2 vaccination in the Tuscany region, 83% of subjects had received one dose of vaccine in the previous six months, 3% at the end of 2022, and 1.8% in April 2023 in the 14–19 age group. Based on data from the Ministry of Health, the vaccine coverage with the second/single dose as of 24 September 2023, among the population aged 12 to 19 in Friuli-Venezia Giulia was 75.63%, in Puglia 83.88%, in Tuscany 82.25%, and in Veneto 76.32%, respectively [29].

## 4. Discussion

Screening conducted in Phase 2 of the pandemic (during 2021–2022) involved 24.4% of the total students enrolled in experimental schools. In the extended Phase 2.0 (2022–2023), the overall participation showed a reduction to 3.5% of students enrolled. During pandemic Phase 2 of this study, the obligation to have a green pass, the introduction of distance learning and the consequent need to carry out a swab for returning to class, as well as the high spread of the virus, influenced the enrolment of the target population, already affected by surveillance for carrying out daily activities. The population under study showed reticence in accepting the screening program despite the meetings organized in each class to communicate the project aims. This downward trend in adherence suggests a potential reduction in motivation or perceived necessity among students and families as the pandemic evolved and public health measures shifted. The 20% drop in reported tests was due to insufficient sample size. In some cases, students failed the test because they were self-administered; in other cases, probably they administered it inappropriately or returned it incomplete as testing positive would have required them to quarantine.

In our study, SARS-CoV-2 swabs were performed every 15 days on all participants to identify positive cases early in the population group under 19 years of age. A meta-analysis by Chen et al. [30] evaluated the detection accuracy of SARS-CoV-2 antigen tests stratified by days after symptom onset and sample type in children and adolescents. The findings demonstrated that the use of nasal swab specimens exhibited high sensitivity for SARS-CoV-2 detection within 7 days after symptom onset. Ejima et al. [31] demonstrated that the average incubation time of SARS-CoV-2 was estimated to be approximately 6 days, and 97.5% of cases developed symptoms in approximately 13 days. These findings confirmed the efficacy of the rotation plan of the screening, performed every 15 days in our study. Considering the emerging spread of new variants of SARS-CoV-2 in the period considered, the asymptomatic spread of SARS-CoV-2 infection among children and adults had considerable relevance.

In parallel to the active screening conducted in the experimental schools, the project made a data collection platform available to the monitoring schools to record the positive COVID-19 cases notified to the school by parents.

Comparing the incidence of the virus in the two populations studied, some critical issues emerge related to the drop in notifications of COVID-19 cases, contextual to the reduction in the spread of the virus. The findings showed a progressive decrease in COVID-19 cases over time, similar to what was observed in the general population.

Nonetheless, during Phase 2.0 (the school years 2022–2023), the COVID-19 cases recorded in schools are identified mainly through screening, while in control schools, the curve remains constant at very low values. Overall, 381 students (2.3%) tested positive for SARS-CoV-2 in control schools in Phase 2.0, with an infection peak recorded between January 2022 and March 2022, while 6.6% (N = 17) of the adherent students tested positive in the experimental school in the same period. That demonstrate the efficacy of this method in identifying SARS-CoV-2 asymptomatic subjects. In addition, the risk of a positive swab was higher in the experimental schools compared to control schools. Waggoner et al. [32] conducted a cross-sectional study of 197 symptomatic children and adolescents aged 4 to 14 years to determine the ability to self-collect nasal swabs for SARS-CoV-2 of the school-aged children testing compared with collection by healthcare workers. The results showed that out of 196 participants, 44.4% were positive for SARS-CoV-2, and 53.6% were negative by self- and healthcare worker-collected swabs. Therefore, after hearing and seeing simple instructional materials, children and adolescents closely agreed on SARS-CoV-2 detection through swabs collected by healthcare workers. Earlier in the pandemic, Thomas et al. [33] in a screening study with throat and nasal swabs involving 40 Western Australian schools showed zero positive test results for SARS-CoV-2, including no false positives.

In our study, the peaks of infections detected through screening coincide with the winter season, as reported in the general population, or with schools reopening after the summer holidays.

In Italy, once the pandemic emergency phase was over, in parallel with the abolition of surveillance tools such as swabs and green passes, students and families significantly reduced the execution of standard screening, mainly resorting to self-administered methods, with consequent under-reporting of the phenomenon. Furthermore, the participation of students and families in the screening program was influenced by the continuous and unpredictable evolution of the epidemic context, together with the development of vaccines, which have helped reduce the risk of SARS-CoV-2 infection. Indeed, vaccination for children aged 12 to 15 also began at the end of May 2021. Comirnaty was the first vaccine authorized in this age group; it had already been approved for adults and adolescents aged 16 and over [34]. According to data from the Ministry of Health [29], the vaccine coverage by a second or single dose as of 24 September 2023, ranged from 75.63% to 83.88% among subjects aged 12 to 19 years in the regions participating in our study.

From the perspective of our study, all this represented a selection bias that strongly influenced the number of cases reported on the platform, both for experimental and control schools. Furthermore, it is essential to underline that the identification of positive cases in control schools presents some missing results due to the failure to register cases in the monitoring platform. Furthermore, participants were enrolled voluntarily in this study, so the screened population may be subject to selection bias; for example, those who felt most at risk of testing positive may not have participated probably because of the fear of having to face quarantine. In addition, in these respects, this study lacks information to compare participants with the entire school population.

## 5. Conclusions

The poor perception of risk and probably the fear of quarantine in case of test positivity significantly influenced participation. The fear of lockdown affected not only the students but also their families, who in many cases, chose not to provide consent for participation in this study. This was because a positive test result could also impact the parents’ daily lives, limiting their ability to work or carry out essential activities.

Furthermore, the large number of government-imposed surveillance measures during that period placed additional stress on young people, testing their resilience and capacity to cope with ongoing restrictions. However, despite the limited participation of the student population in the active screening campaign during the extension phase of the project, this tool showed its effectiveness in the early identification of positive cases. This finding underscores the continued utility of targeted screening initiatives even when adherence is suboptimal. The ability to detect infections at an early stage made it possible to promptly isolate cases and potentially limit further transmission within the school environment. Moreover, the detection of new cases during this phase highlights that, although reduced, the circulation of the virus had not been eliminated and remained a latent risk for the school community.

## Figures and Tables

**Table 1:** Number of enrolled schools per participating region—years 2021–2023.

Regions	Experimental Schools	Control Schools
FVG *	4 secondary schools	8 secondary schools
Veneto	4 secondary schools	8 secondary schools
Tuscany	3 secondary schools	6 secondary schools
Apulia	5 secondary schools	10 secondary schools
Total	16 secondary schools	32 secondary schools

* FVG: Friuli-Venezia Giulia. Legend: Number of secondary schools enrolled in the project by participating region, divided into experimental and control groups, for the years 2021–2023.

**Table 2:** Number of students participating in the screening and incidence of positive cases—analysis by region and total—Phase 2 (October 2021–May 2022).

Region	Total Enrolled Students	Total Enrolled with at Least 1 Valid Swabs	% Positives Out of Total Enrolled	% Positives Out of Total Enrolled with at Least 1 Valid Swab
Friuli-Venezia Giulia	355	329	3.9	4.3
Apulia	182	175	10.4	10.9
Tuscany	222	153	2.7	3.9
Veneto	1474	1432	2.2	2.2
Total	2233	2089	3.2	3.4

Legend: Number of students participating in the screening and incidence of positive cases, by region and total, during Phase 2 of the project (October 2021–May 2022). The table reports the total number of enrolled students, those with at least one valid swab, and the percentage of positive cases calculated both on the total enrolled and on those with valid swabs.

**Table 3:** Number of students participating in the screening and incidence of positive cases—analysis by region and total—Phase 2.0 (September 2022–March 2023).

Region	Total Enrolled Students	Total Enrolled with at Least 1 Valid Swab	% Positives Out of Total Enrolled	% Positives Out of Total Enrolled with at Least 1 Valid Swab
Friuli-Venezia Giulia	105	101	8.6	8.9
Apulia	148	120	4.7	5.8
Tuscany	2	2	0.0	0.0
Veneto	39	36	2.6	2.8
Total	294	259	5.8	6.6

Legend: Number of students participating in the screening and incidence of positive cases, by region and total, during Phase 2.0 of the project (September 2022–March 2023). The table shows the total number of enrolled students, those with at least one valid swab, and the percentage of positive cases calculated both on the total enrolled and on those with valid swabs.

## Data Availability

The original contributions presented in this study are included in the article. Further inquiries can be directed to the corresponding authors.
